# The association between the seasonality of pediatric pandemic influenza virus outbreak and ambient meteorological factors in Shanghai

**DOI:** 10.1186/s12940-020-00625-7

**Published:** 2020-06-17

**Authors:** Yanbo Li, Xiaofang Ye, Ji Zhou, Feng Zhai, Jie Chen

**Affiliations:** 1 University of British Columbia, Vancouver, Canada; 2 Shanghai Key Laboratory of Meteorology and Health, Shanghai Meteorological Service, Shanghai, China; 3 Department of Otolaryngology, Shanghai Children’s Medical Center, affiliated to Shanghai Jiaotong University School of Medicine, 1678 Dongfang Road, Shanghai, 200127 China

**Keywords:** Ambient meteorology, Seasonality, Pediatric pandemic influenza, Influenza virus

## Abstract

**Background and objectives:**

The number of pediatric patients diagnosed with influenza types A and B is increasing annually, especially in temperate regions such as Shanghai (China). The onset of pandemic influenza viruses might be attributed to various ambient meteorological factors including temperature, relative humidity (Rh), and PM_1_ concentrations, etc. The study aims to explore the correlation between the seasonality of pandemic influenza and these factors.

**Methods:**

We recruited pediatric patients aged from 0 to 18 years who were diagnosed with influenza A or B from July 1st, 2017 to June 30th, 2019 in Shanghai Children’s Medical Centre (SCMC). Ambient meteorological data were collected from the Shanghai Meteorological Service (SMS) over the same period. The correlation of influenza outbreak and meteorological factors were analyzed through preliminary Pearson’s r correlation test and subsequent time-series Poisson regression analysis using the distributed lag non-linear model (DLNM).

**Results:**

Pearson’s r test showed a statistically significant correlation between the weekly number of influenza A outpatients and ambient meteorological factors including weekly mean, maximum, minimum temperature and barometric pressure (*P* < 0.001), and PM_1_ (*P* < 0.01). While the weekly number of influenza B outpatients was statistically significantly correlated with weekly mean, maximum and minimum temperature (*P* < 0.001), barometric pressure and PM_1_ (*P* < 0.01), and minimum Rh (*P* < 0.05). Mean temperature and PM_1_ were demonstrated to be the statistically significant variables in the DLNM with influenza A and B outpatients through time-series Poisson regression analysis. A U-shaped curve relationship was noted between the mean temperature and influenza A cases (below 15 °C and above 20 °C), and the risks increased for influenza B with mean temperature below 10 °C. PM_1_ posed a risk after a concentration of 23 ppm for both influenza A and B. High PM_1_, low and the high temperature had significant effects upon the number of influenza A cases, whereas low temperature and high PM_1_ had significant effects upon the number of influenza B cases.

**Conclusion:**

This study indicated that mean temperature and PM_1_ were the primary factors that were continually associated with the seasonality of pediatric pandemic influenza A and B and the recurrence in the transmission and spread of influenza viruses.

## Introduction

Seasonal pandemic influenza, attributable to both types A and B, is particularly prevalent in temperate regions [29]. The onset of influenza viruses is usually characterized by an explicit and predictable annual winter epidemic as a sharp contrast with a less distinct pattern of sporadic outbreaks throughout the tropical regions [8]. Three to five million severe influenza-related illness is reported annually, leading to 250,000–500,000 deaths each year, affecting 20–30% of children and 5–10% of adults worldwide [16, 29, 34]. Young children are one of the most vulnerable groups associated with the highest morbidity and mortality as they are more prone to infections from lacking both prior exposure and immunity to the virus [13].

Previous studies have revealed the correlation between influenza outbreak and ambient meteorological risk factors such as temperature and relative humidity (Rh). A time-series study comparing the incidence of influenza to three climatic parameters including the mean temperature, Rh, and rainfall, in five different cities found an association between the Rh and the incidence of influenza A, and a key association between the mean temperature and the incidence of influenza, in most cities [31]. This corresponded with another study, which showed similar results on the dependence of the dynamics of influenza transmission (or pathology) on temperature and Rh under controlled experimental conditions [19]. Another study indicated that specific humidity plays a significant role in the seasonal transmission of influenza viruses, with two types of environmental conditions, “cold-dry” and “humid-rainy,” being identified as associated with seasonal influenza [30]. However, the relationships between the incidence of influenza and more ambient meteorological factors such as accumulative precipitation, barometric pressure, wind speed and PM_1_ concentrations remained to be elucidated. These factors, together with temperature and Rh, are the most common and standard parameters for measuring atmosphere and climate by the meteorological instrumentation. Barometric pressure is strongly associated with temperature as a low temperature usually indicates high-pressure zone and vice versa. Seasonal variation of wind speed and aerosol concentrations like PM_1_ may influence the influenza seasonality. Specifically, wind dispersion (of the atmospheric aerosols) and the transport by ambient fine particulate matter may significantly affect transmission and concentrations of the airborne influenza virus, and hence increases the exposure risk of the population to the virus [7, 11]. Shanghai features a subtropical monsoon system with higher wind speeds in early spring and autumn, a low subtropical pressure system in summer and a high-pressure system in winter. In this highly polluted city, a high level of atmospheric particulate matter pollution is much more commonly observed in late autumn and winter than summer. It is therefore worthy to investigate how the ambient climate conditions correlate with the influenza seasonality in Shanghai, thereby providing more insights into the interactions between influenza and future climate change.

This study focused on establishing the relationship between the seasonality of pediatric pandemic influenza (A and B) and ambient meteorological factors. By understanding the etiopathogenesis of influenza in relation to ambient meteorological factors, appropriate public health interventions could be implemented to control, mitigate or prevent the outbreak of influenza epidemics among young children.

## Material and methods

### Patients

In this study, we recruited pediatric patients aged from 0 to 18 years who were diagnosed with influenza A or B for over 2 years from July 1st, 2017 to June 30th, 2019 in Shanghai Children’s Medical Centre (SCMC).

### Meteorological data collection

Ambient meteorological data including weekly mean temperature, maximum and minimum temperature, Rh, minimum Rh, atmospheric pressure, wind speed, accumulative precipitation and PM_1_ concentrations were collected from Shanghai Meteorological Service (SMS).

### Statistical analysis

The outpatient and ambient meteorological data from mid-2017 to mid-2019 were sorted and categorized according to weeks, thereby allowing for preliminary simple correlation analysis (Pearson’s r) and the further time-series Poisson regression analysis of non-linear and delayed exposure-response relationship using the distributed lag non-linear model (DLNM). All the ambient meteorological factors as independent variables (IVs) of the Pearson’s correlation were taken into the evaluation of r and their associated *P* values for further non-linear and delayed exposure-response analysis. IVs that were statistically significant were incorporated into the DLNM with a significance level of 0.05 (α = 0.05). Factors with probability lower than the critical level (*P* < 0.05) were accepted and taken into account of the model.

Two separate DLNMs were developed for separate analyses of the relationship with weekly influenza A and B outpatients, and the potential effects from the only time-varying confounding variable which is seasonality (expressed as year and month in the formula) were minimized by controlling this effect moderator in our models. We chose the degree of freedom (df) of 4 for the ambient meteorological factors and a maximum lag of 7 days associated with these factors for the best model fitting based on the Akaike Information Criterion (AIC), and 7 lag days were also determined since the study was based on a weekly analysis. The reference level was defined at the median values of weekly mean temperature and PM_1_ concentrations to calculate the relative risks (RRs) and associated 95% confidence intervals (CIs). The extreme effects of high values and low values were determined at 95th and 5th percentiles respectively with reference to the median values. Specifically, the RRs from high temperature (hot effects) for the DLNM of influenza A and high PM_1_ concentrations (high PM_1_ effects) for the DLNMs of both influenza types were calculated by comparing the 95th percentile to the median values. The RRs and CIs from low temperature (cold effects) for the DLNMs of influenza A and B were calculated by comparing the 5th to the median values. To estimate the cumulative effects (or RRs) of extreme values, a maximum lag of 7 days was also selected for all extreme effects including high and low temperatures, and high PM_1_ concentrations.

All results with *P* < 0.05 were considered statistically significant. All the above data collection, collation and presentation, were carried out by Microsoft Excel 365, and the statistical analyses (Pearson’s r and DLNM) were conducted by RStudio Version 3.6.0, with DLNM carried out using the ‘dlnm’ package.

## Results

### The morbidity of influenza in children by months

**Fig. 1:**
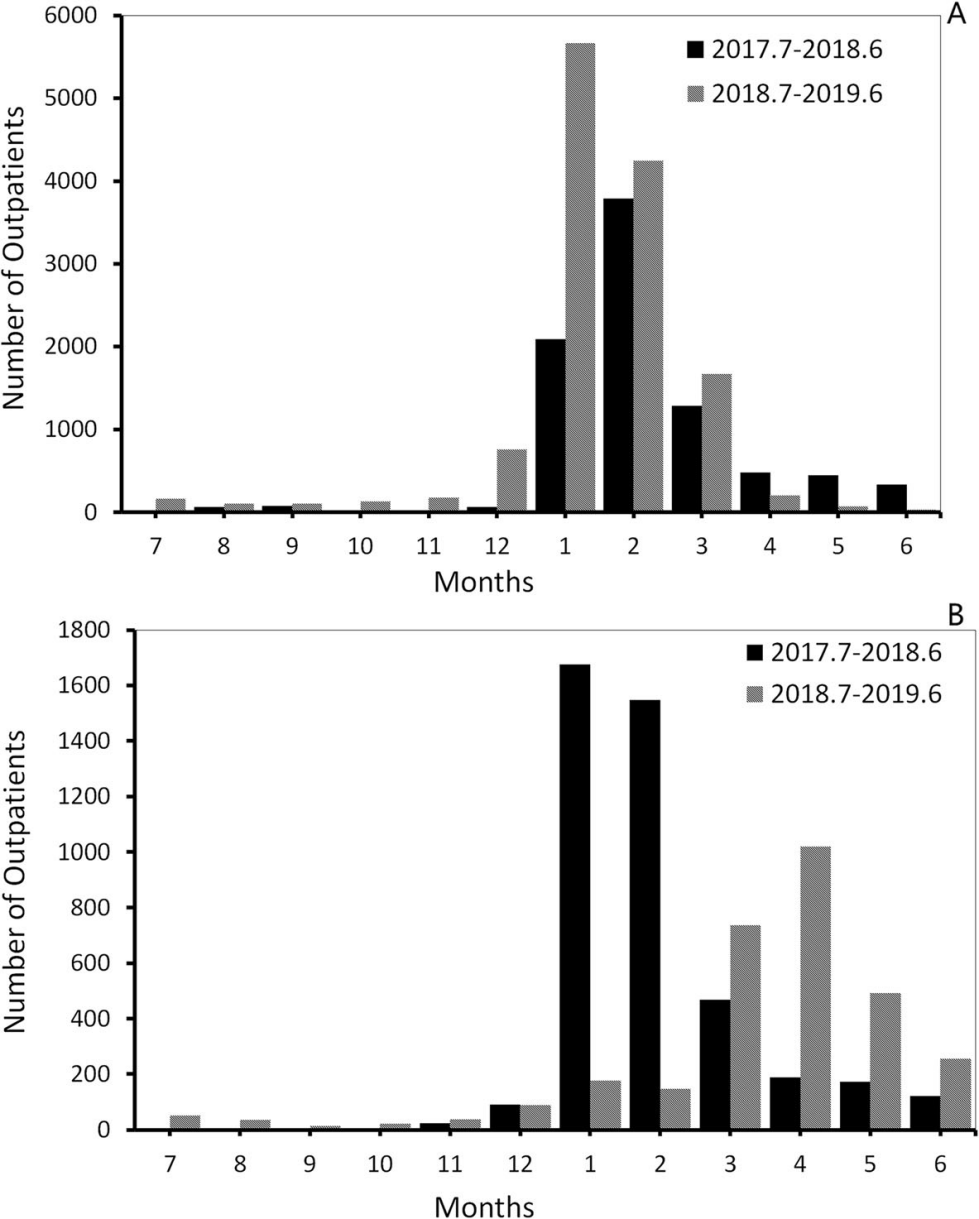
**a** Number of monthly type A outpatients from July 1st, 2017 to June 30th, 2019. **b** Number of monthly type B outpatients from July 1st, 2017 to June 30th, 2019

As shown in Fig. 1a, there was a significant increase in the monthly number of type A outpatients from mid-2017 to mid-2019, particularly in winter (December–February), where a larger peak was observed in 2018–19 than 2017–18.

As shown in Fig. 1b, a slight decline in the monthly number of type B outpatients, was observed from mid-2017 to mid-2019. A winter peak or high-morbidity period was observed from 2018 January to February. There was a delayed effect (or response lag) to the ‘supposed’ winter outbreak of influenza B viruses in 2018–19. Specifically, a relatively smaller peak was noted in spring (March to May) in 2019, compared with the larger winter peak from 2018 January to February. Compared to type A (Fig. 1a), the overall number of type B outpatients was significantly lower.

### The morbidity of influenza in children by age and sex

The number of male and female pediatric type A outpatients from July 1st, 2017 to June 30th, 2019, were 12,196 and 9740, respectively, with a ratio of 1.25: 1. Male children from 0 to 6 years accounted for 43.6% of the total outpatient populations. The highest number of outpatients was seen in the age group of 0 to 3 years, followed by 4 to 6 years, implying that preschoolers and early school-aged children were more susceptible to the infections caused by influenza A viruses than school-aged children (Table. 1).

**Table 1:** Number of type A outpatients from July 1st, 2017 to June 30th, 2019 by age and sex

**Type A**	**2017**		**2017 total**	**2018**		**2018 total**	**2019**		**2019 total**	**Total**
	**M**	**F**		**M**	**F**		**M**	**F**		
0–3 years	53	32	85	2354	1780	4134	2473	2033	4506	8725
4–6 years	43	35	78	2381	1800	4181	2254	1949	4203	8462
7–12 years	29	22	51	739	647	1386	1611	1283	2894	4331
13–18 years	1	5	6	76	55	131	182	99	281	418
**Total**	126	94	220	5550	4282	9832	6520	5364	11,884	21,936

The number of male and female pediatric type B outpatients from July 1st, 2017 to June 30th, 2019, were 4102 and 3277 respectively, with a ratio of 1.25: 1. Male children from 0 to 6 years accounted for 37.9% of the total outpatient populations. The highest number of outpatients was noted in the age group of 4 to 6 years, followed by 0 to 3 years, implying that preschoolers and early school-aged children were more susceptible to the infections caused by influenza B viruses than school-aged children (Table. 2).

**Table 2:** Number of type B outpatients from July 1st, 2017 to June 30th, 2019 by age and sex

**Type B**	**2017**		**2017 total**	**2018**		**2018 total**	**2019**		**2019 total**	**Total**
	**M**	**F**		**M**	**F**		**M**	**F**		
0–3 years	40	18	58	1002	732	1734	349	270	619	2411
4–6 years	17	11	28	919	796	1715	468	369	837	2580
7–12 years	28	15	43	483	398	881	676	583	1259	2183
13–18 years	4	1	5	47	44	91	69	40	109	205
**Total**	89	45	134	2451	1970	4421	1562	1262	2824	7379

### Correlation analysis

#### Influenza A outpatient and ambient meteorological factors

Pearson’s r correlation test was carried out between the number of influenza A and B outpatients and ambient meteorological factors.

As shown in Table. 3, weekly mean temperature, along with maximum and minimum temperature, exhibited a significant negative correlation (r) with the weekly number of influenza A outpatients (*P* < 0.001). Weekly mean barometric pressure and PM_1_ concentrations, on the contrary, showed significant positive correlations (*P* < 0.001 and *P* < 0.01 respectively) with influenza A outpatients. However, there was no significant correlation between influenza A outpatients and weekly mean Rh, minimum Rh, wind speed and accumulative precipitation (*P* > 0.05).

**Table 3:** Pearson’s r test for correlation between weekly influenza A outpatient and statistically significant ambient meteorological factors

**Meteorological factors**	***r***	***P***
Weekly mean T (°C)	−0.62	< 0.001
Weekly mean T_max (°C)	−0.62	< 0.001
Weekly mean T_min (°C)	−0.61	< 0.001
Weekly mean air pressure (hPa)	0.49	< 0.001
Weekly mean PM_1_ (μg/m^3^)	0.25	< 0.01

#### Influenza B outpatient and ambient meteorological factors

As shown in Table. 4, weekly mean temperature, along with maximum and minimum temperature, exhibited a significant negative correlation (r) with the weekly number of influenza B outpatients (*P* < 0.001). Both barometric pressure and PM_1_ concentrations also showed significant positive correlations (*P* < 0.01), while the weekly mean minimum Rh showed moderately significant negative correlation (*P* < 0.05). However, there was no significant correlation between influenza B outpatients and weekly mean Rh, wind speed and accumulative precipitation (*P* > 0.05).

**Table 4:** Pearson’s r test for correlation between weekly influenza B outpatient and statistically significant ambient meteorological factors

**Meteorological factors**	***r***	***P***
Weekly mean T (°C)	−0.46	< 0.001
Weekly mean T_max (°C)	−0.43	< 0.001
Weekly mean T_min (°C)	−0.47	< 0.001
Weekly mean air pressure (hPa)	0.27	< 0.01
Weekly mean rh_min (%)	−0.21	< 0.05
Weekly mean PM_1_ (μg/m^3^)	0.30	< 0.01

The degree to which the weekly number of influenza B outpatients and ambient meteorological factors were correlated (Table. 4) was lower and less discernible than the case of influenza A as shown in Table. 3. Additionally, barometric pressure was excluded from the probability range of *P* < 0.001 (Table. 4), which differed from the case of influenza A (Table. 3).

### Scatter plot

**Fig. 2:**
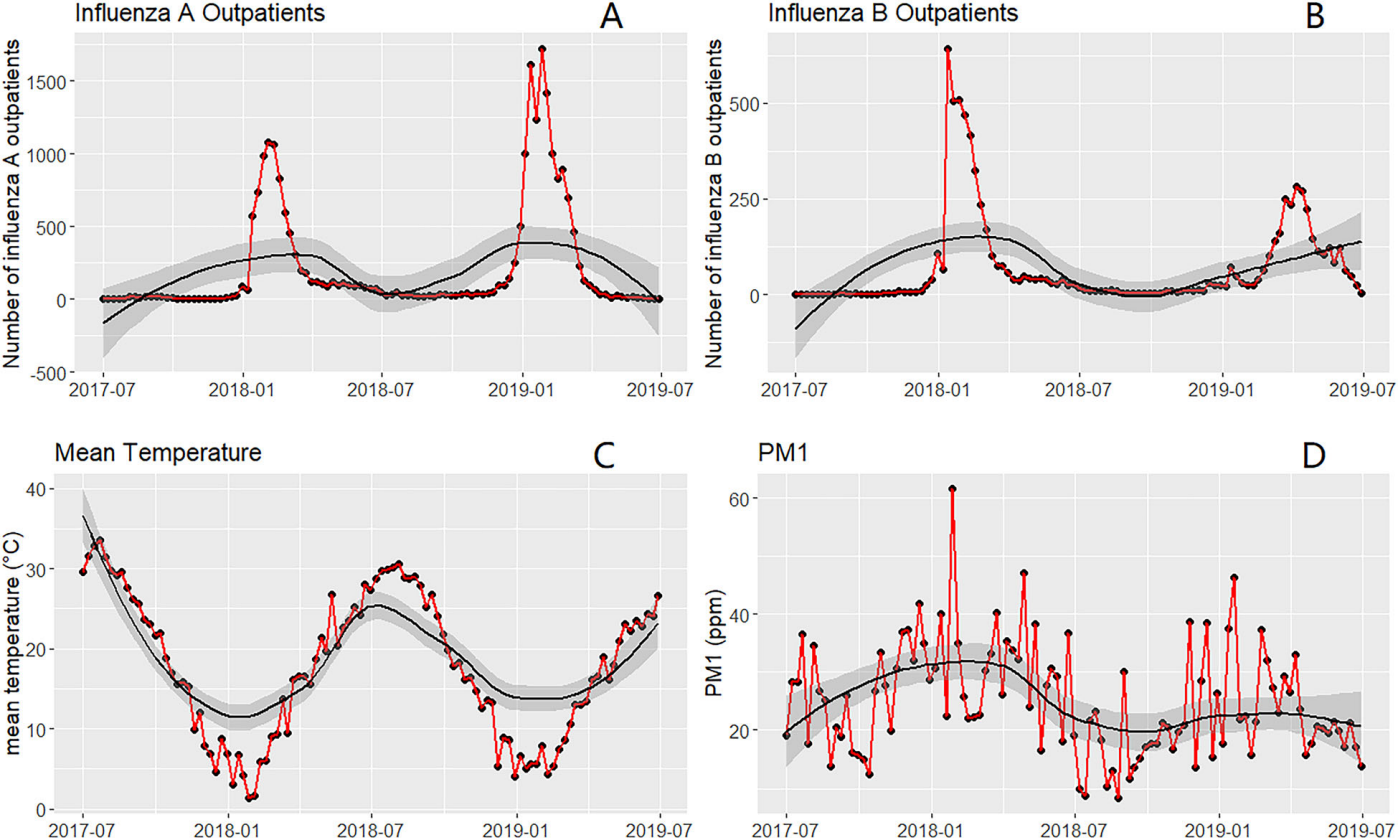
**a**-**d** Scatter plots of influenza A and B outpatients and weekly mean temperature and PM_1_ concentrations

Only statistically significant variables that were common to both types of influenza (aka Tables 3 and 4) were included in Fig. 2 (black lines represent smoothed conditional means, grey regions represent 95% CIs, and solid dots represent weekly values) and DLNMs for subsequent non-linear and delayed regression analysis. Additionally, strong collinearity was also observed in the correlation test among all three temperature-related factors, specifically 0.99 between mean and maximum or minimum temperature, followed by 0.97 between the maximum and minimum temperature. This was also true for barometric pressure as perfect negative collinearity of − 0.92 was noted with mean temperature. Hence, only weekly mean temperature and PM_1_ were included in subsequent models for analysis. Since the weekly number of influenza A and B outpatients followed a Poisson distribution, and the ambient meteorological factors including mean temperature and PM_1_ had lag effects, DLNMs were used in analyzing the effects of these factors on the incidence of seasonal influenza.

### The effects of mean temperature and PM_1_ on pediatric influenza seasonality

**Fig. 3:**
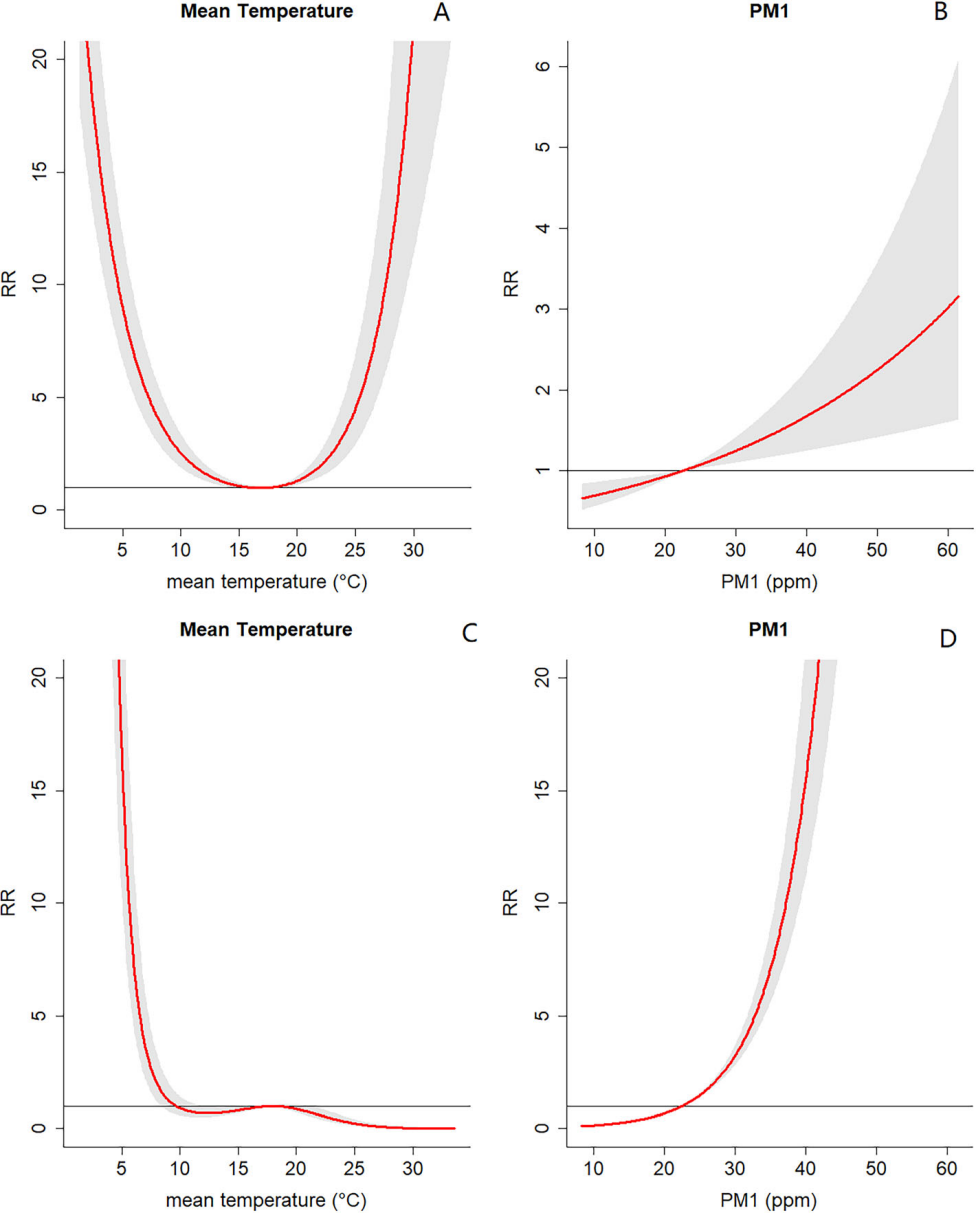
**a**-**b** Estimated overall effects of weekly mean temperature and PM_1_ on the pediatric influenza A cases. **c**-**d** Estimated overall effects of weekly mean temperature and PM_1_ on the pediatric influenza B cases

For an effective visual interpretation, the RRs and 95% CIs of weekly influenza outpatient numbers were plotted against the covariates of mean temperature and PM_1_ for both influenza types, relative to the median values of these meteorological factors and over the corresponding lag days (Fig. 3a-b). For the case of influenza A, the relationship between mean temperature and influenza outpatient numbers was non-linear and could be interpreted as a U-shaped curve (Fig. 3a). The RRs increased with temperatures below 15 °C and above 20 °C, with a continuously higher rate of change either below or above these thresholds (Fig. 3a). A positive relationship was observed between PM_1_ and influenza A cases as there were no known RRs or levels of danger until 23 ppm which was the breakpoint for the onset of influenza incidence risks, in which the RRs increased at a higher rate with higher PM_1_ concentrations (Fig. 3b). For influenza B, the RRs increased below 10 °C (no risks above), with a more significant increase below 5 °C (Fig. 3c). Similar to influenza A, a positive relationship was also observed between PM_1_ and influenza B cases as the RRs also increased above 23 ppm of PM_1_, and there were no risks below the breakpoint (Fig. 3d).

**Fig. 4:**
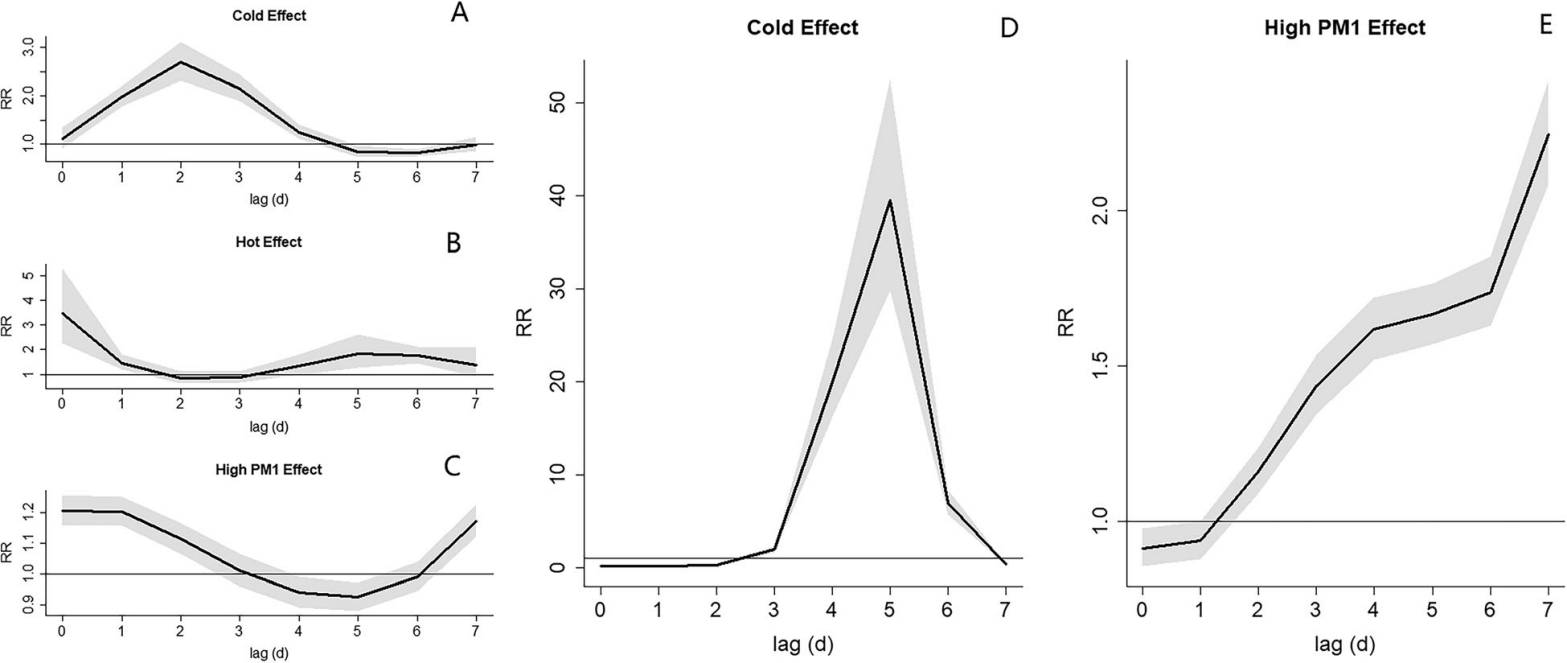
**a**-**c** Lagged cold and hot, and high PM_1_ effects on the pediatric influenza A cases. **d**-**e** Lagged cold and high PM_1_ effects on the pediatric influenza B cases

To identify the cumulative extreme effects on influenza A cases, the estimated effects of mean temperature comparing the 5th and 95th percentile to the median value and PM_1_ comparing 95th percentile to the median value were plotted against lag days in Fig. 4a-c. To identify the cumulative extreme effects on influenza B cases, the estimated effects of mean temperature comparing only 5th percentile to the median value, and PM_1_ comparing 95th percentile to the median value were plotted against lay days in Fig. 4d-e. As shown in Fig. 4a-c, all cold, hot and high PM_1_ were significant and thus considered as risk factors associated with the seasonal occurrence of pediatric influenza A. Cold and high PM_1_ effects were also significant and risk factors associated with the seasonality of pediatric influenza B as shown in Fig. 4d-e. Since all data were analyzed weekly, the maximum lag days selected for the calculation of cumulative extreme effects (or cumulative RRs) of meteorological factors were all 7 days.

We also calculated the cumulative extreme effects of meteorological factors including mean temperature and PM_1_ on pediatric influenza A and B cases (Tables 5 and 6). In terms of influenza A, for the total population of all age children, the RRs of cold and hot effects at 7th lag day were 10.55 (95% CI: 7.81, 14.27) and 22.20 (11.55, 42.68) comparing 5th and 95th percentiles to the median, respectively. The RR of the high PM_1_ effect at the 7th lag day was 1.64 (95% CI: 1.22, 2.19). For influenza B, the RR of cold effect at 7th lag day was 29.74 (95% CI: 18.36, 48.15), and the RR of high PM_1_ effect at 7th lag day was 14.55 (95% CI: 10.55, 20.07). Generally, the cumulative extreme effects of mean temperature were greater than the extreme effects of PM_1_ for both types of influenza (Tables 5 and 6). Additionally, the cumulative extreme effects of both mean temperature and PM_1_ were higher for the case of influenza B than A (Tables 5 and 6). However, the factor of mean temperature showed no cumulative extreme hot effects in Table. 6 since there were no risks above 10 °C (Fig. 3c). Overall, all cumulative extreme effects were statistically significant with *P* ≤ 0.05.

**Table 5:** The cumulative extreme effects of mean temperature and PM_1_ on influenza A incidence at lag day 7

**Meteorological factors**	**Mean temperature (°C)**		**PM**_**1**_**(ppm)**
	**Cumulative extreme cold effects (95% CI)**	**Cumulative extreme hot effects (95% CI)**	**Cumulative extreme high PM**_**1**_**effects (95% CI)**
All age children (0–18 years)	10.55 (7.81, 14.27)	22.20 (11.55, 42.68)	1.64 (1.22, 2.19)

**Table 6:** The cumulative extreme effects of mean temperature and PM_1_ on influenza B incidence at lag day 7

**Meteorological factors**	**Mean temperature (°C)**	**PM**_**1**_**(ppm)**
	**Cumulative extreme cold effects (95% CI)**	**Cumulative extreme high PM**_**1**_**effects (95% CI)**
All age children (0–18 years)	29.74 (18.36, 48.15)	14.55 (10.55, 20.07)

## Discussion

The pediatric influenza outpatient trend from July 1st, 2017 to June 30th, 2019 by time series, age and sex (Fig. 1a-b; Tables 1 and 2) conformed to the general trend of pediatric influenza morbidity. Winter (December/January to March) is the prevailing season for the outbreak of influenza epidemics, whereas April to November is the low-morbidity season of influenza viruses [12, 26]. There was a response lag to the outbreak of influenza B in 2019 winter as delayed to March–May due to differences in antigenic changes and transmission rates between influenza types A and B [1, 33]. Influenza A viruses are more likely to have an antigenic mutation and more efficient transmission than B viruses [1, 33], leading to higher pediatric morbidity and mortality [24, 27, 33]. The sex ratio of the number of male to female pediatric outpatients aged 0–18 was 1.25: 1 for both influenza types. Preschoolers (0–3 years) and early school-aged children (4–6 years) were more susceptible to the infections caused by influenza viruses than older (school-aged) children, which is consistent with findings from other studies [6, 7, 15, 17, 18, 21, 35, 37]. Preschoolers (including infants) are particularly susceptible to influenza virus infections during the epidemic season due to lack of immunity and incapability of their lungs to fight off virus infections, which causes widespread hospitalizations in children diagnosed with influenza A or B [7, 15, 21, 35]. Additionally, school-aged children play a critical role in the transmission of influenza viruses by attending school or daycare, which would lead to greater exposure to adverse ambient meteorological factors and hence the more efficient spread of epidemics [6, 14, 15, 17, 18, 35].

The results from Pearson’s r test revealed an evident negative correlation between the weekly number of pediatric influenza outpatients and corresponding weekly mean temperature, along with maximum and minimum temperature for both influenza types, which has been widely reported [3, 12, 15, 33, 35, 36]. Hence, ambient temperature (mean, maximum and minimum) was closely related to the seasonality of pediatric pandemic influenza A and B [15]. Barometric pressure and PM_1_ concentrations exhibited a weak positive correlation with the weekly number of pediatric influenza A and B outpatients (with the addition of weak negative correlation from between the weekly number of influenza B outpatients and corresponding weekly mean minimum Rh). The barometric pressure was highly negatively correlated with mean, maximum and minimum temperature, as shown by their respective Pearson’s r correlation coefficients–(0.92, − 0.92 and − 0.91), which plays a vital role in the spread and transmission of influenza viruses [15]. Other studies also reported the positive correlation between the number of pediatric influenza outpatients and pollutant levels including PM_2.5_, PM_10_ and O_3_ concentrations as the pollutants could affect the spread of viruses [7, 35], but they did not mention the relationship between PM_1_ concentrations and pediatric influenza incidence, suggesting a potential research deficit on the aspect. Moreover, influenza seasonality was highly associated with minimum Rh [15, 25, 30, 33] as proved by a negative correlation with the number of influenza B outpatients which supports the findings from one study conducted in Beijing [3]. However, the negative correlation contradicts with findings from other studies as minimum Rh was regarded positively correlated with influenza infections [15] due to loss of infectivity under low minimum Rh [10, 11, 15, 23]. Overall, the correlation coefficients between the number of pediatric influenza B outpatients and ambient meteorological factors (Table. 4) were lower and less distinct (or discernible) than the case of influenza A (Table. 3) due to difference in virologic basis including the accumulation of surface antigenic changes and transmission rates [1, 33].

The results and findings from the DLNMs also confirmed that the influenza seasonality was attributed to the effects of ambient meteorological factors including mean temperature and PM_1._ Specifically, we found both lower (below 5 °C) and higher temperature (above 25–30 °C) (a U-shaped curve relationship) were strongly associated with a higher incidence of pediatric influenza A, which corresponds to two previous studies [5, 33]. One study conducted in Jiangsu province, China, which is also a temperate region, indicated influenza A peaked at − 4 °C and 28 °C respectively [5]. Another study conducted in two subtropical regions and one temperate region including Shanghai, Hong Kong, and British Columbia respectively found a similar consistent relationship with influenza incidence peak at low and high temperature in all three regions [33]. A negative relationship between mean temperature and pediatric influenza B incidence was observed as the RR increased significantly below 5 °C, which is consistent with other previous studies [5, 9, 20, 22, 28]. One study in Jiangsu explicitly linked the mean temperature to the occurrence of influenza B by indicating a peak in pediatric influenza B incidence at 5 °C [5]. Other studies from Guangzhou (a subtropical region in SE China) and Seoul (a temperate region in Korea) reported similar findings that there was a significant increase in influenza incidence below 20 °C and 0–5 °C respectively [9, 22], along with a study using a guinea pig model which also explored a link between an increase in influenza transmission rate and temperature below 5 °C [20]. However, no relationship was found in the high-temperature range in these studies [9, 20, 22, 28], except the one in Jiangsu [5]. Apart from mean temperature, we also found a significant increase in PM_1_ concentrations higher than 23 ppm, which is particularly prevalent in winter. Due to limited literature on the non-linear and delayed exposure-response relationship between PM_1_ concentrations and pediatric influenza incidence, we have not yet found supports of this finding from other studies. Regarding the cumulative extreme effects at 0–7 lag days, we found the cumulative RRs were significantly higher in the relationship with influenza B than A as the RR of cumulative extreme cold effects was 29.76 for influenza B, compared with 10.55 for A. The RR for high PM_1_ effects was 14.55 for B, compared with 1.64 for A. Additionally, the effects from mean temperature were more significant and accentuated as a higher increase in cumulative RRs of influenza A and B was caused by temperature (10.55 and 22.20 for cold and hot effects on influenza A respectively, and 29.74 for cold effects on influenza B), compared with PM_1_ (1.64 for high concentration effects on influenza A, and 14.55 for high concentration effects on influenza B). Hence, under simulated multi-factor climatic conditions, both mean temperature and PM_1_ are the two significant primary factors in increasing the risks of seasonal pediatric influenza (A and B) onset and incidence. The epidemiology of influenza was deemed to be the result of the interactions between multiple ambient meteorological factors with temperature and PM_1_ being the two most important contributing factors in driving the seasonal outbreak of influenza pandemics among 0–18 years children.

Overall, these findings provided a useful insight into the seasonal influenza in relation to the current atmospheric setting which is highly uncertain given what the future climate change can hold. There have been several prior research studies investigating the relationship between climate change and the frequency of seasonal influenza onset [2, 4, 32], but only one research indicated a higher likelihood of an early onset of severe seasonal influenza pandemics (commonly known as the flu seasons) for both influenza A and B following warmer than average winters due to larger fraction of susceptible populations being left in the face of a potential outbreak in the next colder season than during normalcy [32]. This specific finding corroborated with one of ours that pediatric influenza patients were more susceptible during winter when the temperature is low, suggested a winter epidemic among children. In the realm of climate change and global warming, it could be anticipated that more severe flu seasons would occur given the increasing frequency of ‘warmer’ winters. Other studies were still uncertain on the exact and more detailed effects of climate change on the seasonality influenza pandemics [2, 4]. Hence, it is certain that the current findings from this study will change in future ‘climate change’ scenario but uncertain regarding the process of changes that would occur given the rising frequency of anthropogenic climate change and associated global warming due to the enhanced greenhouse effect.

## Conclusion

Our study suggested that mean temperature and PM_1_ were continually associated with the seasonality of pediatric pandemic influenza A and B and recurring in the transmission and spread of influenza viruses. Large-sample and multi-centre research are required to obtain a more comprehensive understanding of the interactions between these meteorological variables and the seasonal morbidity of pediatric pandemic influenza.

## Data Availability

The datasets used and/or analysed during the current study are available from the corresponding author on reasonable request.

## Abbreviations

SMS: Shanghai Meteorological Service
SCMC: Shanghai Children’s Medical Centre
Rh: Relative humidity
DLNM: Distributed lag non-linear model
RR: Relative risk
CI: Confidence interval
